# Supplementary material for: In-Depth Longitudinal Comparison of Clinical Specimens to Detect SARS-CoV-2
Source: Pathogens. 2021 Oct 21;10(11):1362. doi: 10.3390/pathogens10111362 (PMC8622859; doi:10.3390/pathogens10111362)
Supplement: Supplementary file 1 [file pathogens-10-01362-s001.zip › pathogens-1383064-supplementary.pdf]

Supplementary material

Table S1. Effect of time on viral load (CT values) in each sample specimen.

Sampling	Intercept	Slope (time effect)
NP	28.0	0.34
Pool	29.1	0.28
TW	30.6	0.23
S	31.9	0.23
OP	32.8	0.20

Table S1 Linear mixed-effect model of CT value with respect to time, sampling methods and their interaction. The sampling methods are sorted by decreasing slope.

Table S2. Comparison of the agreement between NP and TW (Cohen's kappa coefficient) at T1

		TW		
		**Negative**	**Positive**	**Total**
**NP**	**Negative**	4 (5.9)	7 (10.3)	11 (16.2)
	**Positive**	15 (22.1)	42 (61.8)	57 (83.8)
	**Total**	19 (27.9)	49 (72.1)	68 (100.0)

Table S3. Effect of rhinorrhea on the probability of detecting SARS-CoV-2 at T1

Swab	Categories	Total N	Covariate: Rhinorrhea No (n = 51)		Covariate: Rhinorrhea Yes (n = 22)		Rhinorrhea = Yes				
			N	N (%)	N	N (%)	Coeff	SE	OR	95% CI	*p* value
TW			50		21		1.59	0.80	4.89	1.02–23.53	0.047
	Negative	19		17 (34.0)		2 (9.5)					
	Positive	52		33 (66.0)		19 (90.5)					
NP			49		19		−0.94	0.68	0.39	0.10–1.48	0.17
	Negative	11		6 (12.2)		5 (26.3)					
	Positive	57		43 (87.8)		14 (73.7)					
Pool			35		10		−0.94	0.84	0.39	0.07–2.03	0.26
	Negative	8		5 (14.3)		3 (30.0)					
	Positive	37		30 (85.7)		7 (70.0)					
S			46		20		0.12	0.58	1.13	0.36–3.52	0.83
	Negative	21		15 (32.6)		6 (30.0)					
	Positive	45		31 (67.4)		14 (70.0)					
OP			48		18		−0.11	0.56	0.89	0.30–2.66	0.84
	Negative	28		20 (41.7)		8 (44.4)					
	Positive	38		28 (58.3)		10 (55.6)					
R			22		6		12.18	210.7	12.3	/	0.21*
	Negative	27		22 (100.0)		5 (83.3)					
	Positive	1		0 (0.0)		1 (16.7)					

Table S4. Effect of fever on the probability of detecting SARS-CoV-2 at T1

Swab	Categories	Total N	Covariate: Fever No (n = 57)		Covariate: Fever Yes (n = 16)		Fever = Yes				
			N	N (%)	N	N (%)	Coeff	SE	OR	95% CI	*p* value
TW			55		16		1.99	1.07	7.30	0.89–59.66	0.064
	Negative	19		18 (32.7)		1 (6.2)					
	Positive	52		37 (67.3)		15 (93.8)					
NP			53		15		1.18	1.09	3.25	0.38–27.71	0.28
	Negative	11		10 (18.9)		1 (6.7)					
	Positive	57		43 (81.1)		14 (93.3)					

Swab	Categories	Total N	Covariate: Fever				Fever = Yes				
			No (n = 57)		Yes (n = 16)						
			N	N (%)	N	N (%)	Coeff	SE	OR	95% CI	*p* value
S			51		15		2.20	1.07	9.03	1.1–74.13	0.041
	Negative	21		20 (39.2)		1 (6.7)					
	Positive	45		31 60.8)		14 (93.3)					
Pool			34		11		−0.037	0.90	0.96	0.16–5.65	0.97
	Negative	8		6 (17.7)		2 (18.2)					
	Positive	37		28 (82.4)		9 (81.8)					
OP			52		14		0.6	0.62	1.43	0.42–4.85	0.57
	Negative	28		23 (44.2)		5 (35.7)					
	Positive	38		29 (55.8)		9 (64.3)					
R			21		7		12.34	255.1	9.92*	/	0.25*
	Negative	27		21 (100.0)		6 (85.7)					
	Positive	1		0 (0.0)		1 (14.3)					

Table S5. Effect of sore throat on the probability of detecting SARS-CoV-2 at T1

Swab	Categories	Total N	Covariate: Sore throat				Sore throat = Yes				
			No (n = 58)		Yes (n = 15)						
			N	N (%)	N	N (%)	Coeff	SE	OR	95% CI	p value
TW			56		15		1.89	1.07	6.63	0.81–54.41	0.078
	Negative	19		18 (32.1)		1 (6.7)					
	Positive	52		38 (67.9)		14 (93.3)					
NP			57		11		−0.17	0.86	0.84	0.16–4.57	0.84
	Negative	11		9 (15.8)		2 (18.2)					
	Positive	57		48 (84.2)		9 (81.8)					
Salivary			52		14		0.66	0.71	1.94	0.48–7.86	0.35
	Negative	21		18 (34.6)		3 (21.4)					
	Positive	45		34 (65.4)		11 (78.6)					
Oropharynx			54		12		0.95	0.72	2.59	0.63–10.61	0.19
	Negative	28		25 (46.3)		3 (25.0)					
	Positive	38		29 (53.7)		9 (75.0)					
Pool			40		5		−2.35	1.03	0.095	0.01–0.72	0.023
	Negative	8		5 (12.5)		3 (60.0)					
	Positive	37		35 (87.5)		2 (40.0)					
